# Using an Improved Residual Network to Identify PIK3CA Mutation Status in Breast Cancer on Ultrasound Image

**DOI:** 10.3389/fonc.2022.850515

**Published:** 2022-05-26

**Authors:** Wen-Qian Shen, Yanhui Guo, Wan-Er Ru, Cheukfai Li, Guo-Chun Zhang, Ning Liao, Guo-Qing Du

**Affiliations:** ^1^ Department of Ultrasound, Guangdong Provincial People’s Hospital, Guangdong Academy of Medical Sciences, Guangzhou, China; ^2^ Department of Ultrasound, The Second Affifiliated Hospital of Harbin Medical University, Harbin, China; ^3^ Department of Computer Science, University of Illinois Springfield, Springfield, IL, United States; ^4^ College of Medicine, Shantou University, Shantou, China; ^5^ Department of Breast Cancer, Guangdong Provincial People’s Hospital, Guangdong Academy of Medical Sciences, Guangzhou, China

**Keywords:** breast cancer, gene mutation, PIK3CA, deep learning, ultrasonic image

## Abstract

**Background:**

The detection of phosphatidylinositol-3 kinase catalytic alpha (PIK3CA) gene mutations in breast cancer is a key step to design personalizing an optimal treatment strategy. Traditional genetic testing methods are invasive and time-consuming. It is urgent to find a non-invasive method to estimate the PIK3CA mutation status. Ultrasound (US), one of the most common methods for breast cancer screening, has the advantages of being non-invasive, fast imaging, and inexpensive. In this study, we propose to develop a deep convolutional neural network (DCNN) to identify PIK3CA mutations in breast cancer based on US images.

**Materials and Methods:**

We retrospectively collected 312 patients with pathologically confirmed breast cancer who underwent genetic testing. All US images (n=800) of breast cancer patients were collected and divided into the training set (n=600) and test set (n=200). A DCNN-Improved Residual Network (ImResNet) was designed to identify the PIK3CA mutations. We also compared the ImResNet model with the original ResNet50 model, classical machine learning models, and other deep learning models.

**Results:**

The proposed ImResNet model has the ability to identify PIK3CA mutations in breast cancer based on US images. Notably, our ImResNet model outperforms the original ResNet50, DenseNet201, Xception, MobileNetv2, and two machine learning models (SVM and KNN), with an average area under the curve (AUC) of 0.775. Moreover, the overall accuracy, average precision, recall rate, and F1-score of the ImResNet model achieved 74.50%, 74.17%, 73.35%, and 73.76%, respectively. All of these measures were significantly higher than other models.

**Conclusion:**

The ImResNet model gives an encouraging performance in predicting PIK3CA mutations based on breast US images, providing a new method for noninvasive gene prediction. In addition, this model could provide the basis for clinical adjustments and precision treatment.

## 1 Introduction

Breast cancer has become the leading cause of global cancer incidence in 2020 (1), and it is the fifth cause of cancer deaths among Chinese women (2). A high degree of heterogeneity can be observed in breast cancer, and genomic instability is regarded as a major driver of tumor heterogeneity (3). The differences at the genetic and molecular levels make clinical treatment options hugely different. Somatic mutations are stable mutations and play an important role in cancer development and progression (4). The phosphatidylinositol-3 kinase catalytic alpha (PIK3CA) gene is one of the most frequent somatic mutations in breast cancer. According to the Cancer Genome Atlas Network, the percentage of PIK3CA mutations is 34% (5). Phosphatidylinositol 3-kinase (PI3K) is an activator of AKT, which participates in the regulation of cell growth, proliferation, survival, and motility. The PI3K heterodimer consists of two subunits: the regulatory subunit (P85) and the catalytic subunit (p110). PIK3CA induces hyperactivation of the alpha isoform (p110α) of PI3K and can act on the PI3K-AKT-mTOR signaling pathway to trigger oncogene activation, and also lead to persistent AKT activation and regulation of tumor growth in breast cancer (6–8).

Currently, available treatment options for breast cancer are chemotherapy, endocrine therapy (ET), targeted therapy, and immunotherapy. Two-thirds of breast cancer patients express hormone receptors (HR) and lack human epidermal growth factor receptor 2 (HER2) overexpression and/or amplification, and for them, ET is the paramount medical treatment (9, 10). However, about 50% of patients eventually develop ET resistance due to several mechanisms, such as the dysregulation of PI3K-AKT-mTOR signaling (11). The orally available α-selective PIK3CA inhibitor, alpelisib, has been approved by the U.S. Food and Drug Administration (FDA) for the treatment and prognosis of patients with HR+/HER2- advanced or metastatic breast cancer (12, 13). In addition, alterations in the PI3K pathway are associated with poor outcomes of targeted therapy in HER2+ breast cancer (14). For triple-negative breast cancer (TNBC), PIK3CA protein expression is significantly associated with improved overall survival and disease-free survival (15). Therefore, the PIK3CA mutation status plays a vital role in determining the optimal treatment choice for breast cancer patients.

Clinically relevant PIK3CA alterations are detected in several biospecimens using different genetic testing techniques including direct sequencing, real-time polymerase chain reaction (PCR), next-generation sequencing (NGS), and analysis of liquid biopsy samples (16). Although these methods for detecting genetic mutations have improved considerably, molecular testing is often time-consuming, operator dependent, and may be limited by inadequate sample availability. In addition, the cost of genetic testing remains too high for patients. Thus, it is necessary to develop noninvasive and efficient methods for estimating PIK3CA mutation status.

Recently, medical images have been employed to identify the gene mutations in different cancers where different images from different modalities such as computerized tomography (CT) and magnetic resonance imaging (MRI). For instance, Weisset al. (17) found that texture analysis on CT images can differentiate the presence of K-ras mutation from pan-wildtype non-small cell lung cancer. Dang et al. (18) used MRI texture analysis to predict p53 mutation status in head and neck squamous cell carcinoma. Meanwhile, texture analysis has been used to assess the relationship between genetic mutations in breast cancer and morphological features of the masses in MRI images. Woo et al. (19) applied texture and morphological analysis in breast MRI images to evaluate TP53 and PIK3CA mutations. Georgia et al. (20) performed texture analysis of breast MRI to predict BRCA-associated genetic risk. However, CT and MRI are relatively expensive, time-consuming, and not available for all patients.

As one of the widely used tools in breast tumor assessment, ultrasound (US) has similar features to assess breast tumors as CT and MRI and also has the advantages of being non-invasive, real-time, and low cost (21). To solve the disadvantages of operator dependence, many deep learning methods have been proposed for US images. Unlike traditional machine learning and radionics methods, a deep convolutional neural network (DCNN), a special type of deep learning, does not require domain experts to select the specific features beforehand. In contrast, it takes the raw medical images as inputs, does not require manually designed features, and can automatically learn features related to classification or segmentation tasks (22). To improve the efficiency of clinical workflows and reduce inter-observer variation, deep learning has already been applied in large datasets of US images for classifying benign and malignant breast tumors (23–25), classifying molecular subtypes of breast cancer (26, 27), predicting breast cancer lymph node metastasis (28–31) and predicting the response of breast cancer to neoadjuvant chemotherapy (32, 33), etc.

Some studies have applied deep learning models to identify TP53 mutations in pancreatic cancer using MRI multi-modal imaging (34), EGFR mutation status of lung adenocarcinoma using CT imaging (35, 36), and KRAS mutations in colorectal cancer using CT imaging (37). However, it remains unclear whether deep learning models can be employed to identify breast cancer gene mutations on US images. This study observed the differences in breast morphology and other features resulting from microstructural changes in PIK3CA mutant of breast cancers, investigated whether the differences could be captured and interpreted by US images, and identified them using an improved residual network (ImResNet).

## 2 Materials and Methods

### 2.1 Materials

This study enrolled 589 female patients with breast cancer who are treated in Guangdong Provincial People’s Hospital between January 2017 and October 2021. To obtain PIK3CA mutation status, all patients submitted their breast tissue samples and blood samples for targeted sequencing to a clinical laboratory accredited by the College of American Pathologists (CAP) and certified by the Clinical Laboratory Improvement Amendments (CLIA). This retrospective study was approved by the Institutional Review Board of Guangdong Provincial People’s Hospital and exempt from obtaining informed consent from patients.

**Figure 1:**
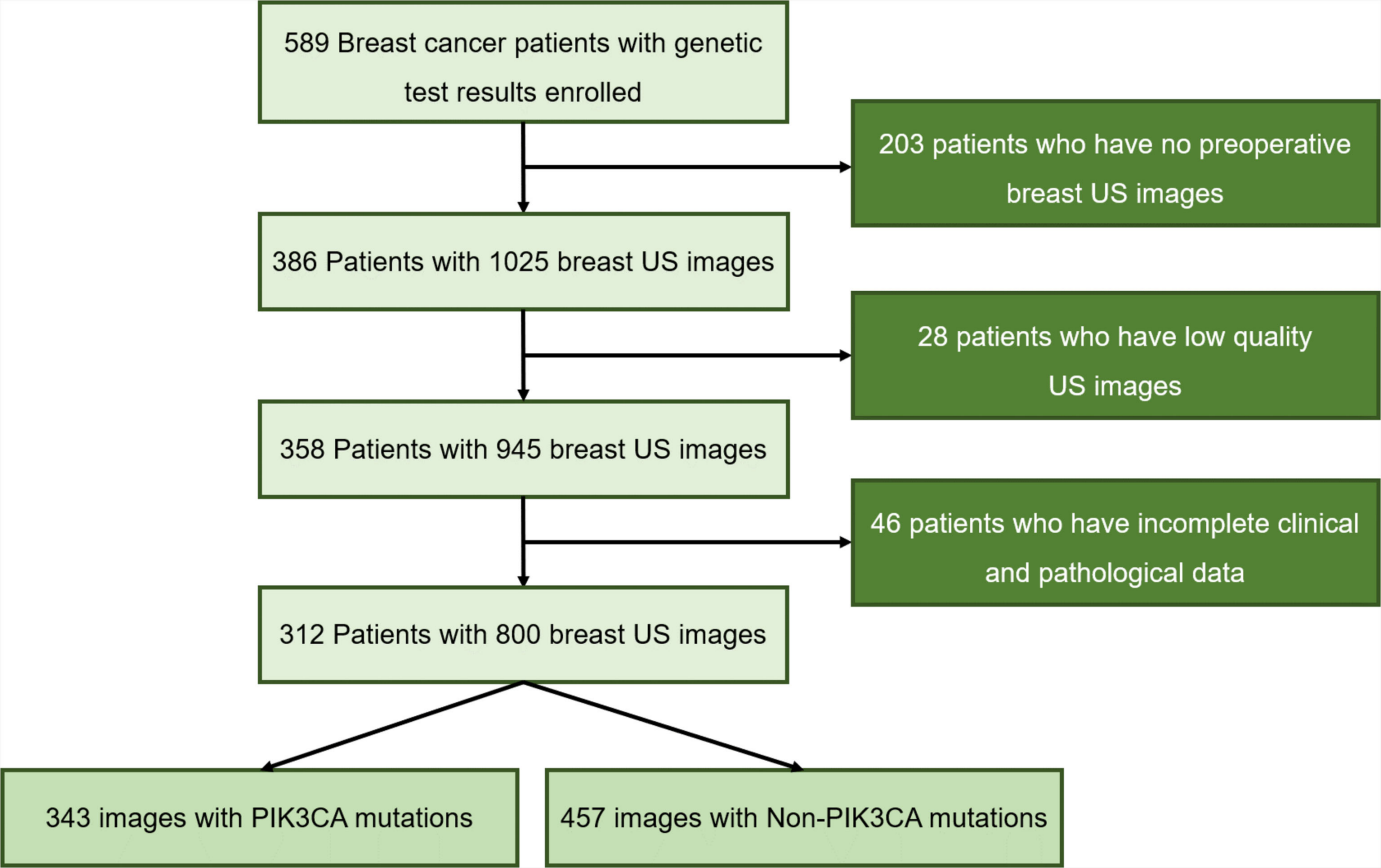
Flowchart of the study cohort selection.

Mutational analysis of the PIK3CA gene was performed using the next-generation sequencing (NGS) technique. First, tissue and genomic DNAs were extracted from formalin-fixed, paraffin-embedded (FFPE) tumor tissues using QIAamp DNA FFPE tissue kit and from blood samples using QIAampDNA blood mini kit (Qiagen, Hilden, Germany), respectively. NGS library construction required at least 50 ng DNA. Then, tissue DNA was sheared using Covaris M220 (Covaris, MA, USA), followed by end repair, phosphorylation, and adaptor ligation. A 200-400bp fragment was purified, followed by hybridization with capture probes decoys, magnetic bead hybridization selection, and PCR amplification. Fragment quality and size were assessed by the high sensitivity DNA kit (Bioanalyzer 2100, Agilent Technologies, CA, USA). Target capture was performed using a commercial panel consisting of 520 cancer-related genes. The cases were selected for study following the criteria ①surgical resection was performed for the target tumor; ②the Pathological and immunohistochemical results were completely obtained; ③the preoperative breast US images of the patients were fully obtained and stored. Finally, 312 patients including 127 PIK3CA mutation patients (the mean age of 51.2 years; the age range of 25-76 years) and 185 Non-PIK3CA mutation patients (the mean age of 48.7 years; the age range of 22-89 years) with 800 US images were collected in this study. The flowchart of the study cohort selection is shown in **Figure 1**. To ensure the robustness and accuracy of the model, multiple US images of different sections were acquired per lesion as much as possible.

### 2.2 Proposed Methods

#### 2.2.1 Tumor Region Extraction

**Figure 2:**
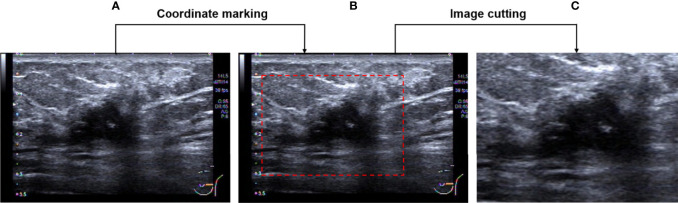
Image pre-processing. **(A)** An original breast US image. **(B)** Image after coordinate marking. **(C)** The selected effective image area.

Firstly, the region of interest (ROI) which includes the entire tumor area, as well as the minimum peritumoral tissue was manually cropped in breast US images which were completed by a senior radiologist with 12 years of experience. An example of the ROI of breast US images is shown in **Figure 2**. After that, a total of 800 ROI images were obtained and then split into training and testing groups at the ratio of 75% to 25%.

#### 2.2.2 Deep Learning Network

In the proposed method, the PIK3CA mutation status is observed on the ROI images and the mutation identification problem is transferred into an image classification problem. A deep residual network (ResNet) is redesigned by changing the architecture to extract the textural features on images and its output parts were modified to accomplish this classification task.

In deep learning networks, multiple layers are stacked in sequence and the output of the previous layer is fed to the following layer. A convolution layer is a basic layer where different filters perform a convolution operation to extract the features from the former layers with different kernels (22). The *k^th^* convolution layer *L_k_* is noted as:


(1) $$x_{n}^{k}=f\left(\sum_{m}x_{m}^{k-1}⊗W_{mn}^{k}+b_{n}^{k}\right)$$

(2) $$f(x)=\{\begin{array}{ll} x & x\geq 0\\ 0 & x<0 \end{array}$$

where $x_{m}^{k-1}$ is the *m^th^* feature map of layer *L_k_*_-1_, $W_{mn}^{k}$ is the connecting weights between *n^th^* feature map of the output layer and *m^th^* feature map of the previous layer, and the bias of *n^th^* feature map is denoted as $b_{n}^{k}$ ⊗ denotes the convolution operation, $W_{mn}^{k}$ is randomly initialized and is then tuned using a backpropagation procedure, and further optimized with stochastic gradient descent (SGD) algorithm (38), *f* is an activation layer to convert the nonlinear values into linear values. There are some commonly used activation functions namely rectified linear units (ReLU), Sigmoid, Tangent and softmax functions (39).

Pooling layers reduce the redundant parameters in the convolution layer to increase the computing speed.


(3) $$I^{\left(k+1\right)}=P(I^{\left(k\right)})$$

Where *P* is a pool function, the max pooling, average pooling, global max pooling, and global average pooling methods are used for this process.

In the fully connected layer, each neuron is connected to the previous layer. Their outputs estimate the confidence to different categories.

For a classification problem, the final layer usually uses an activation function as the classification layer. The classification layer yields the probabilities of the inputs belonging to a certain class (40).


(4) $$P(y=1|x,w,b)=\frac{exp\{w\cdot x+b\}}{1+exp\{w\cdot x+b\}}$$

(5) $$P(y=0|x,w,b)=\frac{1}{1+exp\{w\cdot x+b\}}$$

Where *y* is the class target, *x* ∈ *R* ^Nx1^ is a *N* dimensional feature vector, *w* ∈ *R* ^Nx1^ is the weight parameter, and *b* is a bias term. In our model, the output layer has two outputs for PIK3CA and Non-PIK3CA mutation, respectively.

**Figure 3:**
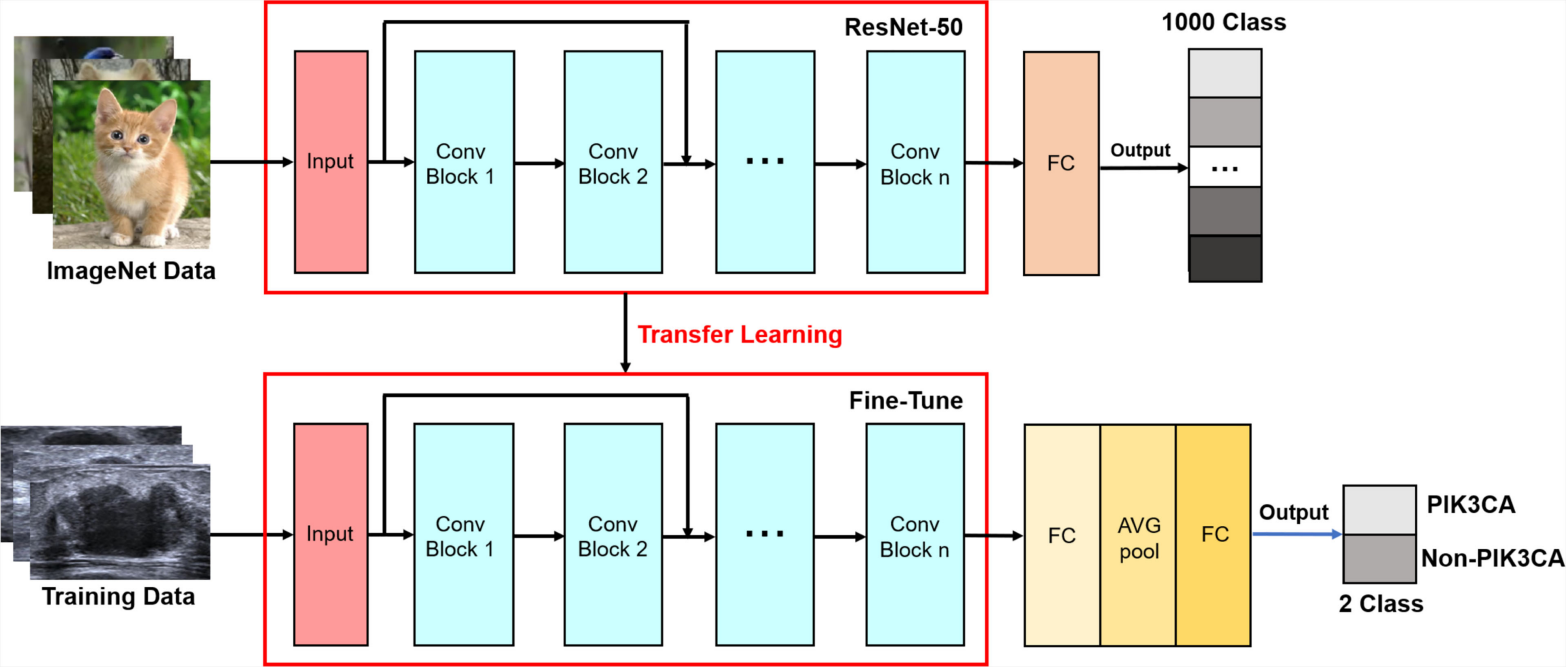
The ImResNet model’s structure diagram.

The ResNet employs a unique residual operation in the network which makes it easy to converge, to gain accuracy from increased depth. A ResNet utilizes skip connections, or short-cuts, to jump over some layers. Typically, it consists of convolutional layers, rectified linear units (ReLU) layers, batch normalization layers, and layer skips (41). The transfer learning approach redesigns the pre-defined network to make it accomplish different tasks, which reduces the time in training and improves the network’s generalization ability. In our proposed network, rather than building a model from scratch, a ResNet50 model pre-trained by natural images from ImageNet, is selected as a backbone to extract the features from ROI images. ImageNet comprises more than 14 million images that have been hand-annotated to indicate the pictured objects and are categorized into more than 20,000 categories (42). Of note, in breast US transfer learning, ImageNet is used as a pre-training dataset in most cases (43–45). The advantages of using the pre-trained network include reducing training time, providing better performance for neural networks, and requiring limited data. The original ResNet is improved by adding a new fully connected layer for feature extraction and adding a new global average pooling to interpret these features in the classification task. The idea is to generate one feature map for each corresponding category of the classification task in the last convolutional layer. Thus, the feature maps can be interpreted as categories confidence maps. Also, the global average pooling is a structural regularize to prevent overfitting for the overall structure. Then, another fully connected layer is added as a classification layer to match the output numbers of classified categories, and a binary cross-entropy (BCE) function is used as the loss function which computes the BCE between predictions and targets (46). **Figure 3** shows the structure diagram of our proposed ImResNet.

### 2.3 Evaluation and StatisticalAnalysis Metrics

A confusion matrix (CM) is used to evaluate classification performance. The rows of CM represent the instances of a predicted class and columns represent the instances of an actual class, Using the results in CM, four parameters namely precision (P), recall (R), F1-score, and accuracy (ACC) were defined as follows:


(6) $$\text{P(i)}=\frac{M_{\text{ii}}}{\sum_{j}M_{ji}}$$

(7) $$\text{R(i)}=\frac{M_{\text{ii}}}{\sum_{j}M_{\text{i}j}}$$

P(i) is the fraction of samples where the algorithm correctly predicted class i out of all predictions using the algorithm, and R(i) is the fraction of cases where the algorithm correctly predicted i out of all the true cases of i. M_ij_ is the samples whose true class is i and prediction class is j.


(8) $$\text{F}1(\text{i})=2\times \frac{\text{P(i)}\times \text{R(i)}}{\text{P(i)}+\text{R(i)}}$$

(9) $$\text{ACC}=\frac{\sum_{i}M_{\text{ii}}}{\sum_{\text{i}j}M_{\text{i}j}}$$

where ∑_i_M_ii_ is all correct predictions and ∑_i_ *_j_* M_i_ *_j_* is total predictions. Accuracy is one metric for evaluating classification performance, which is defined as a fraction of correct predictions out of total predictions.

The receiver operator characteristic (ROC) curve was also utilized to measure the classification performances of different models. The area under curve (AUC) was calculated and worked as a metric to evaluate the classification performance.

## 3 Results

### 3.1 Platform Settings

The modified deep learning model was trained on a server with a 2 x Six-Core Intel Xeon processor and 128GB of memory. The server is equipped with an NVIDIA Tesla K40 GPU with 12GB of memory.

### 3.2 Predictive Performance of the ImResNet Model

**Figure 4:**
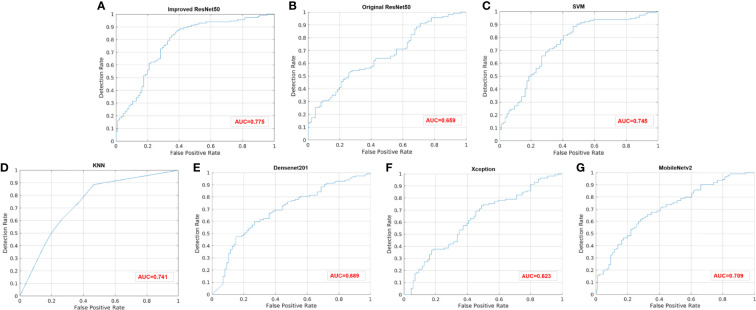
ROC curves of different models. **(A)** Improved ResNet50. **(B)** Original ResNet50. **(C)** SVM. **(D)** KNN. **(E)** DenseNet201. **(F)** Xception. **(G)** MobileNetv2.

**Figure 5:**
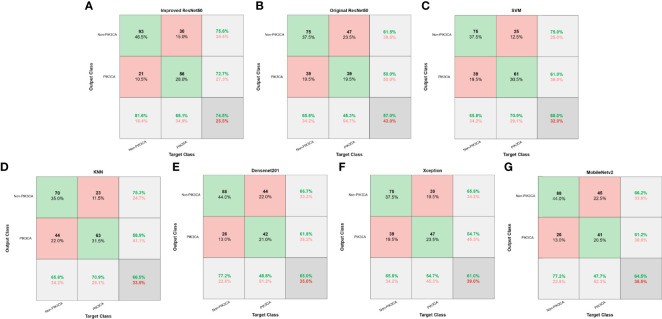
Confusion matrices of different models. **(A)** Improved ResNet50. **(B)** Original ResNet50. **(C)** SVM. **(D)** KNN. **(E)** DenseNet201. **(F)** Xception. **(G)** MobileNetv2.

**Figure 6:**
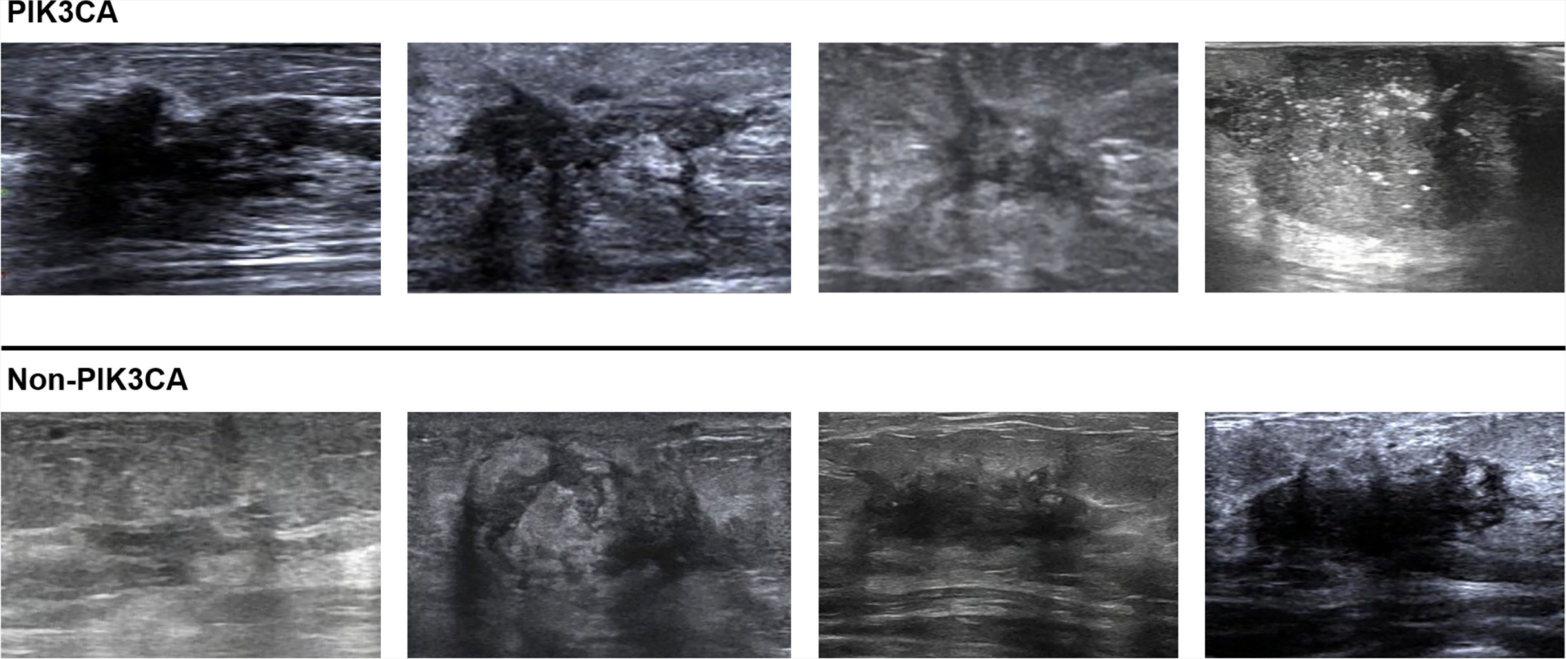
Classification examples of the ImResNet model.

For the test set of 200 US images, the performance of the ImResNet50 model has been given in **Table 1**. The ImResNet model achieved the best performance in all models, with an overall accuracy of 74.50%, and the average precision, recall, and F1-score reached 73.35%, 74.17%, and 73.60%, respectively. **Figure 4A** shows the model achieved an AUC of 0.775. Besides, the performance of the ImResNet model can be visualized from the CM in **Figure 5**. In the figures of CM, the first two rows represent the instances of a predicted class, the first two columns represent the instances of an actual class, the diagonal elements correspond to correctly classified observations, and the off-diagonal cells correspond to incorrectly classified observations. As well, the bottom row is the row-normalized row summary, and it shows the percentages of correctly and incorrectly classified observations for each true class. The rightest column is the column-normalized column summary and displays the percentages of correctly (in green color) and incorrectly classified observations (in red color) for each predicted class. In each cell, the percentage value is calculated using the current number over the whole sample number. **Figure 6** shows the classification examples of the ImResNet model. In the first line, the four images in the PIK3CA category are listed, while the four images in the Non-PIK3CA category are shown in the second row.

**Table 1:** A performance summary of the ImResNet model and other models in identifying PIK3CA mutations of breast cancer.

**Model**	**Classifier**	**Categories**	**Precision**	**Recall**	**F1-score**	**Accuracy**
Machine learning	SVM	Non-PIK3CA	64.04%	74.49%	68.87%	
		PIK3CA	70.93%	59.80%	64.89%	
		Average	67.48%	67.15%	66.88%	
		Overall				67.00%
	KNN	Non-PIK3CA	61.40%	75.27%	67.63%	
		PIK3CA	73.26%	58.88%	65.28%	
		Average	67.33%	67.07%	66.46%	
		Overall				67.33%
Deep learning	ImResNet50	Non-PIK3CA	81.58%	75.61%	78.48%	
		PIK3CA	65.12%	72.73%	68.71%	
		Average	73.35%	74.17%	73.60%	
		Overall				74.50%
	Original ResNest50	Non-PIK3CA	65.79%	61.48%	63.56%	
		PIK3CA	45.35%	50.00%	47.56%	
		Average	55.57%	55.74%	55.56%	
		Overall				57.00%
	DenseNet201	Non-PIK3CA	77.19%	66.67%	71.54%	
		PIK3CA	48.84%	61.76%	54.55%	
		Average	63.02%	64.22%	63.05%	
		Overall				65.00%
	Xception	Non-PIK3CA	65.79%	65.79%	65.79%	
		PIK3CA	54.65%	54.65%	54.65%	
		Average	60.22%	60.22%	60.22%	
		Overall				61.00%
	MobileNetv2	Non-PIK3CA	77.19%	66.17%	71.26%	
		PIK3CA	47.67%	61.19%	53.59%	
		Average	62.43%	63.68%	62.42%	
		Overall				64.50%

### 3.3 Comparison With Machine Learning Models

First, we compared our proposed ImResNet model with two commonly used machine learning methods to identify PIK3CA mutations on the same dataset. In machine learning, the support vector machine (SVM) (47) is one of the most robust supervised learning models for classification and regression analysis, which transfers the training examples to points in space to maximize the width of the gap between the two categories and maps the new unknown examples into that same space and predict their belongings to a category based on which side of the gap they are in. The K-nearest neighbors (KNN) algorithm is a type of instance-based classification method where an unknown object is classified by a plurality vote of its neighbors, with the object being assigned to the class most common among its K nearest neighbors. In the parameters of KNN, 5 neighbors are selected. Euclidean distance is the distance metric, and all features are standardized in the range of [0, 1]. The two machine learning models’ performance is listed in **Table 1**, and the ROC curves are depicted in **Figures 4C, D**. The ImResNet achieves an AUC of 0.775, higher than that of SVM and KNN models (AUC: 0.745, 0.741). The overall accuracy, average precision, recall rate, and F1-score of the ImResNet model were all significantly higher than the SVM and KNN models. The CMs of the two machine learning models are shown in **Figures 5C, D**. We find that compared with the SVM and KNN models, the ImResNet model has an improvement in the ability to identify Non-PIK3CA mutation. Compared with the KNN, the ImResNet model has increased 23(11.5%) correctly identified cases in Non-PIK3CA mutation.

### 3.4 Comparison With Deep Learning Models

To confirm the enhanced performance of the improved ResNest50 model, we compare it with the original ResNest50 model and other deep learning models (DenseNet201, Xception, MobileNetv2). We obtained the ROC curves, AUC values (as shown in **Figure 4**), accuracy, precision, recall, and F1-score (as presented in **Table 1**). Our model’s AUC value was 11.6% higher than original ResNest50, 8.6% higher than DenseNet201, 15.2% higher than Xception, and 6.6% higher than MobileNetv2. Meanwhile, all quantitative metrics are better than other deep learning models. From the CM in **Figure 5**, we found that the ImResNet model has increased 17(19.8%), 5(2.5%), 18(9.0%) and 5(2.5%) correctly identified cases in PIK3CA mutations compared to the original ResNest50 model, DenseNet201, Xception and MobileNetv2, respectively.

## 4 Discussion

In this study, we proposed a DCNN-ImResNet using non-invasive US images to identify PIK3CA mutation status for patients with breast cancer. As one of the most common mutated genes in breast cancer, PIK3CA plays an essential role in both the development and progression of breast cancer (48, 49). As an oral PI3K inhibitor, Alpelisib has received FDA approval for targeted breast cancer therapy (13). Accordingly, determining the PIK3CA mutation status of breast cancer patients is critical to the management. Whereas complexity of genetic testing has limited timely testing and targeted treatment to breast cancer patients in the era of precision medicine. Previously, Woo Kyung et al. (19) found that texture analysis of segmented tumors on breast MRI based on ranklet transform was potential in recognizing the presence of TP53 mutation and PIK3CA mutation, and for PIK3CA mutation, the AUC of ranklet texture feature was 0.70. But this study has some limitations. On the one hand, acquiring MRI images of breasts is time-consuming and expensive. On the other hand, the computer-aided diagnostic approach in that study is semi-automated and still needs manual interactions. Hence, we proposed the ImResNet model which can automatically identify PIK3CA mutations. So far, it is the first study of US images based on deep learning for the identification of PIK3CA mutations in breast cancer.

The ImResNet model is a feasible model for identifying PIK3CA mutations with an AUC of 0.775 for the test cohorts, outperforming the two machine learning models (SVM and KNN) and other deep learning models (Original ResNest50, DenseNet201, Xception, and MobileNetv2). The good performance obtained illustrates that the differences in breast morphology and other features resulting from microstructural changes in PIK3CA mutant breast cancers could be captured by US images and identified using a deep learning model. The ResNet50 has been proven to have good performance in breast US images classification because it is possible to go deeper without losing generalization capability (26). We used transfer learning to pre-train ResNet50 to overcome our small sample size problem and improved the original ResNet50 by adding a new fully connected layer for feature extraction and adding a new global average pooling to interpret these features in the classification task to obtain the ImResNet. Then, we trained the ImResNet model using the presence or absence of the PIK3CA mutations as a label and finally confirmed that the PIK3CA mutation status can be identified from US image data alone.

One of the advantages of our model is that it automatically learns US image features without the need to extract features manually. In recent years, radiomics features extracted from non-invasive images have been applied to identify gene mutations in some tumors. Zhang et al. (35) proposed to develop a deep learning model to recognize EGFR status of LADC by using the radiomics features extracted from CT images. Their results show that this method can precisely recognize EGFR mutation status of lung adenocarcinoma patients. Nevertheless, the radiomics features rely on manual annotation by professionals and automatic segmentation of the target area. Manual annotation is time-consuming and labor-intensive. Moreover, automated segmentation requires a well-established segmentation system in clinical practice. By contrast, deep learning models can automatically learn multi-level features. A study by Kan et al. (37) investigated performance by using a deep learning method to estimate the KRAS mutation status in colorectal cancer patients based on CT imaging and compared it with a radiomics model, and the results show that the deep learning model has a better performance.

Meanwhile, some studies have focused on pathological specimens of tumors to test whether deep learning models can predict gene mutations from pathological pictures. Wang et al. (50) demonstrated that a DCNN could assist pathologists in the detection of BRCA gene mutation in breast cancer. Velmahos et al. (51) used a deep learning model to identify bladder cancers with FGFR-activating mutations from histology images. Furthermore, Nicolas et al. (52) trained a DCNN to predict the ten most commonly mutated genes in lung adenocarcinoma on pathology images. They found that six of them (TK11, EGFR, FAT1, SETBP1, KRAS, and TP53) can be predicted with AUCs from 0.733 to 0.856. However, some histopathological information can only be evaluated after invasive biopsy or surgery resection. The proposed ImResNet model on US images can repeatedly be tracked during the exploration of tumor treatment when the patient’s physical condition is not suitable for invasive biopsy or surgery.

Despite the better performance of the ImResNet model to identify PIK3CA mutations, it still has several limitations that can be improved in future work. First, the sample size was relatively small and retrospectively collected in this study. Therefore, prospective investigation using considerably larger datasets is required to further validate the robustness and reproducibility of our conclusions. Second, we included only a single-center cohort with the internal testing set. In the future, multi-centercohorts should be recruited for evaluation. Third, the 74.50% accuracy of our proposed method is not yet sufficient for clinical needs, and further performance improvements are needed in future work. However, this promising performance could still encourage more researchers to utilize deep learning methods based on US imaging to identify breast cancer gene mutations.

## 5 Conclusion

In this study, we proposed a DCNN-ImResNet for the automated identification of PIK3CA mutations in breast cancer based on US images. Our method’s main advantage is that it is a non-invasive method for identifying PIK3CA mutations in breast cancer suitable for avoiding invasive damage when surgery and biopsy are inconvenient. In addition, US images are easily available to monitor for PIK3CA mutations throughout the treatment period of breast cancer. And the cost and time to obtain US images are relatively low. Although the ImResNet model has some potential in identifying PIK3CA mutations, there is still space for performance improvement. In the future, prospective multicenter validation should be performed to provide a high level of evidence for the clinical application of the ImResNet model.

## Data Availability Statement

The original contributions presented in the study are included in the article/supplementary material. Further inquiries can be directed to the corresponding authors.

## Ethics Statement 

The studies involving human participants were reviewed and approved by The Institutional Review Board of Guangdong Provincial People’s Hospital. The ethics committee waived the requirement of written informed consent for participation.

## Author Contributions

G-QD and NL conceived and designed the study. YG designed the proposed method and accomplished experiments. W-QS, W-ER, CL, and G-CZ collected the clinical and imaging data. W-QS and W-ER formed the data interpretation and the statistical analysis. All authors approved the final manuscript.

## Conflict of Interest

The authors declare that the research was conducted in the absence of any commercial or financial relationships that could be construed as a potential conflict of interest.

## Publisher’s Note

All claims expressed in this article are solely those of the authors and do not necessarily represent those of their affiliated organizations, or those of the publisher, the editors and the reviewers. Any product that may be evaluated in this article, or claim that may be made by its manufacturer, is not guaranteed or endorsed by the publisher.
